# The Effects of Strength Training Combined with Vitamin C and E Supplementation on Skeletal Muscle Mass and Strength: A Systematic Review and Meta-Analysis

**DOI:** 10.1155/2020/3505209

**Published:** 2020-01-08

**Authors:** Maurilio T. Dutra, Wagner Rodrigues Martins, Alexandre L. A. Ribeiro, Martim Bottaro

**Affiliations:** ^1^College of Physical Education, University of Brasilia, 70910-900 Brasilia, DF, Brazil; ^2^Federal Institute of Education, Science and Technology, IFB, Campus Recanto das Emas, 72620-100 Brasilia, DF, Brazil; ^3^School of Physical Therapy, University of Brasilia, 72220-275 Brasilia, DF, Brazil

## Abstract

Intense muscle contractile activity can result in reactive oxygen species production in humans. Thus, supplementation of antioxidant vitamins has been used to prevent oxidative stress, enhance performance, and improve muscle mass. In this sense, randomized controlled studies on the effect of vitamin C and E supplementation combined with strength training (ST) on skeletal muscle mass and strength have been conducted. As these studies have come to ambiguous findings, a better understanding of this topic has yet to emerge. The purpose of the present review is to discuss the current knowledge about the effect of vitamin C and E supplementation on muscle mass and strength gains induced by ST. Search for articles was conducted in the following databases: PubMed/Medline, Web of Science, Scopus, Cochrane Central Register of Controlled Trials, and Google Scholar. This work is in line with the recommendations of the PRISMA statement. Eligible studies were placebo-controlled trials with a minimum of four weeks of ST combined with vitamin C and E supplementation. The quality of each included study was evaluated using the Physiotherapy Evidence Database Scale (PEDro). 134 studies were found to be potentially eligible, but only seven were selected to be included in the qualitative synthesis. A meta-analysis of muscle strength was conducted with 3 studies. Findings from these studies indicate that vitamins C and E has no effect on muscle force production after chronic ST. Most of the evidence suggests that this kind of supplementation does not potentiate muscle growth and could possibly attenuate hypertrophy over time.

## 1. Introduction

Oxidative stress (OS) can be defined as a state of cellular imbalance between the production and the physiological capacity to neutralize reactive species [1]. Current understanding of this phenomenon includes the potential damage that this imbalance may inflict to macromolecules, such as lipids, proteins, and deoxyribonucleic acid (DNA) [2]. Also, decreased control of mitochondrial respiration and decreased integrity of the sarcoplasmic reticulum are known to be OS related [3]. Therefore, it is not a surprise that chronic OS has been found to be associated with the aging process [4, 5], as well with several diseases, such as atherosclerosis, metabolic syndrome, and cancer [6].

The term reactive species encompasses a wide range of chemical substances. Although nitrogen, iron, copper, and sulfur species are encountered, reactive oxygen species (ROS) like hydrogen peroxide (H_2_O_2_), superoxide (O_2_·^−^), and the hydroxyl radical (·OH) are mostly examined [4]. Generally, it has been assumed that an increase in oxygen consumption by mitochondria would lead to an augmented formation of ROS at the electrical transport chain [3]. So, it could be expected that physical exercise would acutely increase ROS production by increasing oxygen consumption. Indeed, scientific evidence from the 80s and 90s demonstrated that acute physical exercise triggers the overproduction of ROS and enhances OS in rats [7, 8]. Nowadays, there is no doubt that intense muscle contractile activity can also result in OS in humans [3]. Plenty of recent evidence shows that acute aerobic [9], anaerobic [10, 11], and strength exercise [12, 13] triggers the production of ROS, even though it is a transient phenomenon.

Based on the evidence of increased OS after a single bout of exercise [14], the interest of scientists and physically active people upon exogenous antioxidant supplementation raised during the last decades. Noteworthy, consumption of antioxidant compounds is widely spread [15] and varies from 16% among recreational exercisers to 50% among elite athletes in the United States of America (USA) [16]. Amateur and professional athletes are particularly interested in preventing the damaging effects of ROS. Antioxidant supplementation could, supposedly, prevent this damage and thereby reduce fatigue and enhance muscle performance with such supplementation [16]. In addition, regular consumption of dietary supplements (including antioxidants) was found to be high among USA college students who wanted to “increase muscle strength” and “enhance performance” [17].

Among the most reported antioxidant supplements in the literature are vitamins C (i.e., ascorbic acid) and E (i.e., tocopherol). Vitamin C is water-soluble and present on the cytosolic compartment of the cells [18]. It is known not only by its antioxidant properties, but also by bone and cartilage maintenance, hormone synthesis, and immunity [19]. Vitamin E is fat-soluble and found in virtually all cell membranes [18]. As a lipophilic antioxidant, vitamin E protects membranes from being oxidatively damaged by ROS. Also, its biological roles include immune function and gene expression [19, 20]. Of note, vitamin C serves as an electrical donor to vitamin E radicals generated in the cell membrane during OS [18]. Moreover, previous results indicate that combined supplementation with vitamins C and E enhances cytokine production in healthy adults and therefore could be more immunopotentiating than supplementation with either vitamin alone [21]. So, some researchers started to seek for understanding the effects of combining vitamin C and E supplementation on exercise-induced OS and adaptations [22, 23]. Interestingly, some previous studies show that these two antioxidant vitamins in combination, or even alone, could acutely reduce markers of OS (i.e., malondialdehyde and protein carbonyls) after aerobic [24] and strength exercise [25].

However, from a chronic perspective, recent investigations have demonstrated that ROS have relevant physiological actions that may upregulate skeletal muscle adaptations, such as mitochondrial biogenesis, induction of antioxidant defense, and hypertrophy [26]. Indeed, it was also demonstrated that antioxidant vitamin supplementation on a regular basis hampers exercise training adaptation in animals [27]. So, a change in the understanding of ROS role in training adaptations is underway. On this matter, research on strength exercise models in humans is scarce and the first published investigations came up with conflicting results. While some reported a positive effect of vitamins C and E on muscle mass and plasma antioxidant status of elderly people [28, 29], others showed negative effects on muscle thickness [30] and bone mineral density [31]. Regarding young people, available findings show that supplementation may actually hamper strength gain [32, 33] or is ineffective [34, 35].

Once strength training (ST) is well known to be a potent stimulus to physiological and performance adaptations (i.e., increase in muscle strength and hypertrophy) [36, 37], a better understanding of the effects of this supplementation strategy combined with ST in a long-term basis has yet to emerge. Moreover, although there are published systematic reviews about the effect of proteins [38] and other vitamins [39, 40] on muscle strength and mass, there is no published systematic review about vitamin C and E effect on ST adaptations. Thus, the aim of this systematic review was to summarize evidence from randomized controlled trials that examined the effect of ST combined with vitamin C and E supplementation on skeletal muscle mass and strength.

## 2. Materials and Methods

### 2.1. Protocol and Registration

The present review was accredited under the number CRD42017078761 in the *International Prospective Register of Systematic Reviews* (PROSPERO). Also, this work is in line with the recommendations of the PRISMA statement to report systematic reviews [41].

### 2.2. Eligibility Criteria

This systematic review was performed according to the PICO acronym: Population: healthy and/or nonhealthy adult and elderly humans; Intervention: strength training combined with vitamin C and E supplementation; Comparator: strength training without supplementation or no intervention; Outcomes: variables related to muscle strength and/or muscle hypertrophy. Eligible studies to compose the present review were placebo-controlled clinical trials with a minimum of four-week duration. Only studies of ST combined with vitamin C and E supplementation were selected, regardless of the vitamin's dosage. In addition, only studies written in English were considered eligible. These criteria ensure the methodological quality of interventions and provide specific information for human performance and sport scientists. Exclusion criteria were animal models' studies, reviews, case reports, books, conference abstracts, and MSc/PhD thesis.

### 2.3. Search and Study Selection

Two independent authors performed an electronic search between 10/30/2018 and 11/27/2018. Electronic search for articles was completed at PubMed/Medline, Web of Science, Scopus, Cochrane Central Register of Controlled Trials, and Google Scholar. Search for full articles was conducted by adopting the following strategy: (“strength training” OR “resistance training” OR “eccentric training”) AND (“antioxidant supplementation” OR “vitamin c” OR “vitamin e” OR “ascorbic acid” OR “alpha tocopherol”). Studies were first carefully analyzed by their titles and abstracts. After exclusion of articles not meeting inclusion criteria, the remaining full texts were critically read and some of them were finally selected to be included in the qualitative synthesis of the present work. Once studies were identified, two independent reviewers screened titles and abstracts for relevance. In cases of disagreement, a third reviewer was consulted. The same reviewers analyzed the full text to determine the studies to be included. The reference list of the articles included was consulted to find possible additional studies. Duplicated items were removed by importing the search results into Mendeley Desktop 1.19.2.

### 2.4. Data Extraction

Data collected were authors, year, study design, population and recruitment, number of participants, age, training description and control groups, assessment protocol, and outcomes. The continuous data to perform the meta-analysis were extracted by one reviewer and checked by a second reviewer. Disagreements were resolved through discussion. The values were entered into a database on Excel Software before the use of Review Manager Software (version 5.3.5).

In order to investigate the effects of supplementation, we planned to perform two analyses: (1) the effects on muscle strength and (2) the effects on muscle hypertrophy. Considering that the few studies included regarding muscle hypertrophy presented different instruments to assess muscle structure (dual energy X-ray absorptiometry, DEXA; ultrasound, US; magnetic resonance imaging, MRI) and different populations (adults and older adults), the meta-analysis for muscle hypertrophy outcome was not employed. Furthermore, one of these studies did not provide the values of the outcome to perform the analysis. We tried to contact the author by email, but we had no response. With respect to muscle strength outcome, considering the included studies that used only the isokinetic equipment to assess muscle strength, we performed one meta-analysis using the mean difference (MD: measures the absolute difference between the mean values in two groups in a clinical trial) and 95% of the confidence intervals were considered [42]. To this analysis, we used changes from baseline, the standard deviation of changes from baseline (SD change) and the number of participants (“*n*”) per group. When a study did not report the data to estimate the SD change, the following equation was used:

(1) $$\begin{array}{c} \text{SD change}=\sqrt{\left({\left[\text{SD pre}\right]}^{2}+{\left[\text{SD post}\right]}^{2}-2\times \text{corr}-\left[\text{pre, post}\right]\times \text{SD pre}\times \text{SD post}\right)}. \end{array}$$

Considering that the included papers had distinct intervention parameters and settings, a random-effects model (interstudy heterogeneity) was always employed in the meta-analysis. Finally, a sensitivity analysis was planned to identify if a specific study (forest plot inspection for outliers) changes the summary effect, by repeating the meta-analysis with one study omitted at a time. The heterogeneity of the studies was assessed by the *I*^2^ statistic and 95% CI [42]. An *I*^2^ statistic 0% to 30% might not be important, 30% to 60% may represent moderate heterogeneity, 50% to 90% may represent substantial heterogeneity, and 75% to 100% may represent considerable heterogeneity [43]. Assessment of clinical relevance was made using three categories: small effect (MD < 10% of the scale; SMD < 0.5); medium effect (MD from 10% to 20% of the scale; SMD from 0.5 to 0.8); large effect (MD > 20% of the scale; SMD > 0.8) [44].

### 2.5. Methodological Quality Assessment

The methodological quality of the identified RCTs was scored using the PEDro scale [45] that presents 11 items (random allocation; concealed allocation; baseline comparability; blind subjects; blind therapists; blind assessor; adequate follow-up; intention-to-treat-analysis; between groups comparisons; point estimates and variability), rated as “yes” or “no.” The first item is not used for score calculation; thus, it ranges from 0 to 10 points. Trials with a PEDro score ≥6 points were classified as high quality, while trials with a PEDro score <6 points were classified as low quality. The assessment of selected studies was performed according to the Brazilian-Portuguese version of the PEDro scale.

## 3. Results

**Figure 1:**
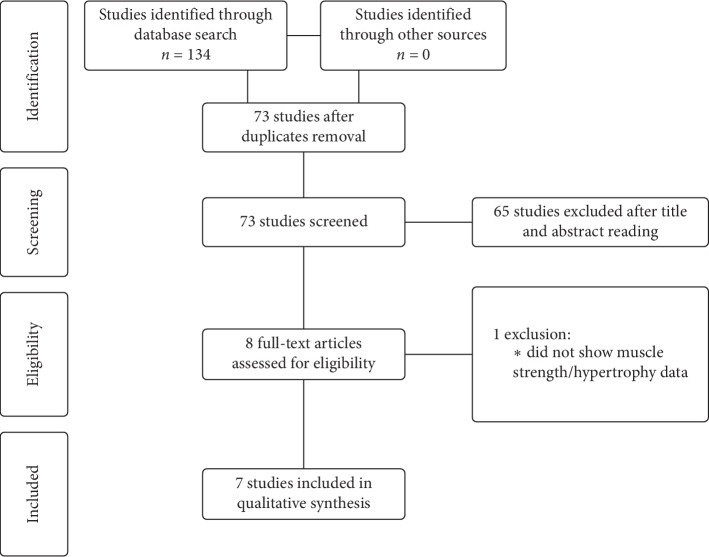
Flowchart of the study.

Database search returned a total of 134 results. Sixty-one duplicated items were removed, and 73 studies were found to be potentially eligible for this review. After reading the titles, 49 studies were excluded (main reasons were usage of other types of supplements, less than four-week designs, acute studies, and aerobic exercise interventions). So, 24 studies were selected for abstract analysis. A critical reading of the abstracts was performed, and 16 studies were removed (9 were literature reviews, letter to the editor, or conference report; other reasons were usage of other antioxidants, lack of supplementation, and lack of muscle strength/hypertrophy analysis). Therefore, 8 articles were fully read and critically analyzed for inclusion. One of these articles was excluded at this stage because it did not present data on muscle strength and hypertrophy. Consequently, seven studies were selected to be critically reviewed and discussed.  Figure 1 presents the flow of the selection of articles of this review.

All articles included in the present review reached scores ranging from eight to ten in the PEDro scale, indicating high methodological quality. Outcomes different from muscle strength and hypertrophy (i.e., insulin sensitivity, lipid profile, and biomarkers of OS) reported in the selected articles were not discussed as they are out of the scope of the present work. The main characteristics and results of the selected articles are summarized in Table 1.

**Figure 2:**
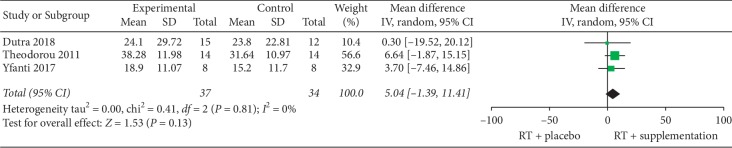
Results of the meta-analysis.

A total of 3 studies (exercise + supplementation, *n* = 37; exercise + placebo, *n* = 34) were included in the meta-analysis of muscle strength (isokinetic equipment): Theodorou et al. [34], Yfanti et al. [35], and Dutra et al. [33]. There were no statistical differences on muscle strength gains between groups (MD = 5.09 N/m; 95% CI: −1.39 to 11.41; *Z* = 1.53; *P*=0.13) (Figure 2).

## 4. Discussion

The present work aimed at reviewing the literature about the effect of ST combined with vitamin C and E supplementation on skeletal muscle mass and strength. Seven studies were included in the qualitative synthesis, but not all of them were included in the meta-analysis due to the methodological reasons already mentioned. The present discussion will first focus on strength gain outcomes and then on muscle hypertrophy.

Regarding the effects of antioxidant supplementation on strength gains, six studies reported data and the results of four of them [28, 30, 34, 35] indicate that vitamin supplementation has no influence on strength gain. Within these six studies, meta-analysis was done with the three studies that employed isokinetic assessment of muscle strength [33–35]. Results of the meta-analysis confirmed that supplementation is innocuous regarding this outcome (Figure 2). In other words, vitamin supplementation was neither positive nor negative. Importantly, the studies evaluated strength in the elderly after traditional [28] or an undulating model of periodization [30] or used isokinetic eccentric training in adult men [34, 35]. Thus, it could be inferred that vitamin C and E supplementation is ineffective with respect to strength gains independent of the population (elderly/adults), and the manipulation of training variables such as type of training (traditional/eccentric), exercises, training frequency, and volume/intensity. Furthermore, Paulsen and colleagues [32] showed that upper body strength gains were significantly higher in the placebo group (+17.1 vs. +7.6 % 1 RM gain) when compared to the supplemented group in a sample of young men and women after ten weeks of a progressive ST program. Overall, these findings indicate that beyond ineffective, vitamin C and E supplementation could even be detrimental to strength gains in a long-term basis. This is the opposite of what could be inferred from acute design studies, as well as from the logic “conclusion” that neutralizing exercise ROS production would be beneficial to performance.

There would be reasons to believe that supplementation could be beneficial, once it is reported in the literature that excessive exposure to ROS could decrease muscle capacity to generate force by affecting myofilament function and sarcoplasmic reticulum calcium regulation, leading to muscle fatigue [46, 47]. However, it is also discussed that moderate amounts of ROS play a positive role in force generation and that there is an optimal cellular redox state in which conditions are ideal for muscle force production [46]. In other words, chronic neutralization of ROS production during RT by supplementation could interfere with this endogenous/physiological balance and reduce ROS production to a level that is innocuous to improve muscle contractile activity. Hence, chronic strength levels are unaffected or even mitigated in response to antioxidant vitamin supplementation.

In the present review, none of the five studies that reported data regarding muscle hypertrophy was included in the meta-analysis, but is also relevant to discuss their results. Since the aging process is related to a physiological excess ROS production [4, 5], it could be hypothesized that antioxidant supplementation would prevent OS in the elderly and boost ST adaptations. In line with this assumption, Bobeuf et al. [29] investigated the effects of vitamin C (1000 mg) and E (600 mg) supplementation combined with ST in a sample of 48 sedentary elders of both sexes. Participants of that study underwent a six-month high-intensity RT program (3 sets, 8 reps, 80% 1 RM). The authors found that only participants who combined ST with supplementation presented fat-free mass gain (+1.5 kg) after the intervention. They discuss that vitamin C and E supplementation likely reduced damage and/or increased protein synthesis induced by muscle contraction associated with ST. However, they did not measure protein oxidation or synthesis. Interestingly, a different result was reported in a subsequent study of Bobeuf et al. [28] adopting the same protocol, but with a larger sample (*n* = 57). The authors observed that not only one but both groups (ST combined with supplementation and ST without supplementation) presented a significant fat-free mass gain after 6 months compared to baseline. Also, they did not observe fat-free mass differences between groups after the intervention. Possibly, the larger sample size in the second study provided more power to the analysis and a more accurate result, as this was the only difference between their studies. In this sense, the recent study of Bjørnsen et al. [30] found that after 12 weeks of an undulating periodized RT program, *rectus femoris* thickness (+3.4 vs. +1.9 mm), as well as legs lean body mass (around 5% vs. 2% gain) of elderly men increased more in the placebo than in the supplemented group. The authors discuss that the use of high-dose antioxidant supplementations to modulate the aging process could suppress physiologically important ROS-mediated actions, such as the activation of hypertrophy pathways. Actually, it has been argued that ROS are signaling molecules to skeletal muscle anabolism [48], and this might be true when moderate amounts of ROS are produced as a consequence of muscle contractile activity. Possible mechanistic explanation relies on muscle gene expression alteration induced by ROS via phosphorylation of kinases and transcriptional activating factors [48]. As a matter of fact, a previous study demonstrated that oral administration of vitamin C attenuated hypertrophy induced by mechanical overload in rats [49]. It seems like excess vitamins C and E may suppress hypertrophy pathways mediated by ROS, such as signaling of ERK1/2 and p70S6K [49] and this is in line with the result reported by Bjørnsen et al. [30]. Yet, it is still complex to explain why they found a result that is in the opposite direction when compared to Bobeuf et al. [29], where only participants who combined ST with supplementation presented fat-free mass gain. Of note, despite the methodological differences in the interventions, Bjørnsen and colleagues found an overall larger muscle mass gain that possibly enabled them to detect the “adverse” effects of the supplementation.

Two studies analyzed muscle mass adaptations in young men and women, and they found no effects of supplementation. Participants from Paulsen et al. [32] were allocated in supplemented (i.e., 1000 mg vitamin C and 235 mg vitamin E) or placebo groups and performed a traditional progressive ST regimen (3 to 4 sets, 6 to 7 exercises, 11 to 6 RM) for 10 weeks. The authors found that cross-sectional areas of upper and lower body muscles increased similarly in supplemented and placebo groups (8.5 vs. 7.6% and 3.8 vs. 3.8%, respectively) after the intervention. In the other study [33] participants were divided into vitamin (i.e., 1000 mg vitamin C and 400 IU of vitamin E), placebo, and control (i.e., no supplementation and no ST) groups. Vitamin and placebo groups performed an ST program (2 to 4 sets, 4 exercises, 12 to 8 RM) for ten weeks. After the intervention, the thickness of the *rectus femoris* muscle increased similarly in placebo (39.7 ± 5.2 to 42.5 ± 5.6 mm) and vitamin (37.2 ± 5.4 to 40.3 ± 5.6 mm) groups. In other words, antioxidant supplementation did not boost muscle hypertrophy and this may be related to the recent and previously mentioned understanding that ROS are, in fact, signaling molecules to muscle adaptation.

There are some different findings (i.e., no effect; positive effect; negative effect) reported in the selected studies of the present review and they cannot be neglected. However, conflicting results may be related to several methodological factors such as differences in periodization schemes, volume/intensity of training, methods of strength and hypertrophy assessment, trial duration, and age of the participants. Basal levels of vitamins and supplementation dosage are as important as ST variables when it comes to the results. In addition, training status of participants and gender are relevant issues to consider. Three of the studies analyzed trained subjects [32, 34, 35]. Importantly, OS in response to strength exercise sessions is minimal in trained subjects [50, 51]. This could be related to the lack of effect of supplementation reported by some articles. Similarly, three studies recruited a mixed sample of men and women [28, 29, 32], while only one recruited only women. As women may present higher basal levels of vitamin E and some other antioxidants [52], it could be possible that gender influences the results of studies that analyzed mixed samples. Nevertheless, the overall analysis and the meta-analysis of the present work allow coming to some relevant conclusions.

In summary, the results of the seven studies indicate that chronic supplementation of vitamins C and E has no positive/ergogenic effect on muscle strength. Furthermore, studies show that it could even be detrimental [32, 33]. Regarding muscle mass gain, most of the evidence suggests that this kind of supplementation does not potentiate skeletal muscle hypertrophy. From the five reviewed studies, only one presented findings in favor of the supplementation [29], while three did not show any differences between groups [28, 32, 33] and one presented a negative effect of the supplementation [30]. It is worthy to note that it was not possible to perform a meta-analysis with regard to muscle hypertrophy outcomes, and only three studies were included in the meta-analysis of muscle strength. This absence of more studies in the meta-analysis may limit the discussion. Yet, the present work certainly adds understanding to the state of the art of the topic.

It is also relevant to mention that evidence is growing on the biological roles of ROS and its cell signaling effects that may result in positive muscle adaptations [48]. Yet, further investigation and critical appraisal of the situations that may warrant vitamin C and E supplementation (i.e., hypovitaminotic cases) in ST are welcome. Of note, recent evidence suggests that antioxidant supplementation can be optimized in exercise contexts when the antioxidant status of each individual is taken into account [53]. Also, the study of different intensities of ST and lower doses of vitamin supplementation combined with the assessment of OS markers and endogenous antioxidant capacity could provide science-based information to athletes, coaches, and recreational trainers who want to enhance skeletal muscle adaptations in a long-term basis.

## Figures and Tables

**Table 1:** Main characteristics and findings of the selected studies.

Study	PEDro score	Sample	Training	Duration	Vitamins	Strength	Hypertrophy
Bobeuf et al. [29]	10	*n* = 48 sedentary men and women (±65.5 y)	3x/wk; 7 exercises; 3 sets, 8 reps; 80% 1 RM; sit-ups included	6 months	600 mg/d vitamin E; 1000 mg/d vitamin C	Data unavailable	Fat-free mass gain (+1.5 kg) only in RT plus supplementation group

Bobeuf et al. [28]	10	*n* = 57 sedentary men and women (±65.3 y)	3x/wk; 7 exercises; 3 sets, 8 reps; 80% 1 RM; sit-ups included	6 months	600 mg/day vitamin E; 1000 mg/d vitamin C	Significant and similar % 1 RM increase in RT (+65.0%) and RT plus supplementation (+78.2%)	Fat-free mass gain NS between RT (+0.3 kg) and RT plus supplementation (+0.5 kg)

Theodorou et al. [34]	9	*n* = 28 trained men (±25.6 y in placebo, ±26.2 y in vitamins)	Isokinetic eccentric; 2x/wk; 5 sets; 15 reps; 60°/s; seated position	11 weeks with 4 weeks of training	1000 mg/d vitamin C; 400 IU/d vitamin E for 11 weeks	Isometric peak torque increased similarly (15% in placebo; 18% in vitamins) in both groups	Data unavailable

Paulsen et al. [32]	9	*n* = 32 trained men and women (±27.0 y in vitamins, ±24.0 y in placebo)	Traditional, progressive ST; 4x/wk; 3 to 4 sets; 6 to 7 exercises; 11 to 6 RM	10 weeks	1000 mg/d vitamin C; 235 mg/d vitamin E	% 1 RM gain in bíceps curl greater in placebo group (+17.1 vs. +7.6); isometric force gain only in the placebo group	CSA of upper (8.5 vs. 7.6%) and lower limbs (both 3.8%) increased similarly in vitamins and placebo groups The same for LBM
Bjørnsen et al. [30]	9	*n* = 34 sedentary elderly men (±68.0 years)	Free weight exercises; weekly undulating periodization; 3x/wk	12 weeks	1000 mg/d vitamin C; 235 mg/d vitamin E	Significant and similar 1 RM increase in placebo and antioxidants groups	*Rectus femoris* thickness increased more in placebo (+3.4 vs. +1.9 mm). The same for LBM (2.2 vs. 0.87 kg)

Yfanti et al. [35]	8	*n* = 16 trained men (±24.6 y in vitamins, ±25.9 y in control)	Isokinetic eccentric; 2x/wk; 5 sets; 2 min rest; 15 reps; 60°/s in seated position	9 weeks with 4 weeks of training	1000 mg/d vitamin C; 400 IU/d vitamin E for 9 weeks	Significant and similar peak torque increase in placebo (+6.6%) and vitamins group (7.9%)	Data unavailable

Dutra et al. [33]	8	*n* = 42 untrained women (±23.7 y in vitamins, ±24.0 y in placebo, ±23.6 y in control)	Traditional, progressive ST; 2x/wk; 2 to 4 sets; 4 exercises; 12 to 8 RM	10 weeks	1000 mg/d vitamin C; 400 IU/d vitamin E for 70 days	Significant peak torque increases in placebo and vitamins. Only placebo was different when compared with control (*P*=0.01)	*Rectus femoris* thickness increased similarly in placebo (39.7 to 42.5 mm) and vitamins group (37.2 to 40.3 mm)

RT: resistance training; y: years; wk: week; CSA: cross-sectional area; LBM: lean body mass; RM: repetition maximum.
